# Slovenian Validation of the Mental Health Literacy Scale (S-MHLS) on the General Population: A Four-Factor Model

**DOI:** 10.1177/00469580211047193

**Published:** 2022-02-08

**Authors:** Nina Krohne, Vanja Gomboc, Meta Lavrič, Tina Podlogar, Vita Poštuvan, Nuša Zdravec Šedivy, Diego De Leo

**Affiliations:** 1 Andrej Marušič Institute, Slovene Centre for Suicide Research, 68960 University of Primorska, Koper, Slovenia; 2 Department of Psychology, Faculty of Mathematics, Natural Sciences and Information Technologies, 244385 University of Primorska, Koper, Slovenia

**Keywords:** mental health literacy, psychometrics, factor analysis, reliability, validity

## Abstract

The aim of the present validation study is to determine the psychometric properties of the Slovenian version of the Mental Health Literacy Scale. For this purpose, the factorial structure, internal consistency, cross-cultural, convergent and discriminant validity were assessed. The measure was translated and adapted to Slovenian context through a blind back-translation process. It was applied to a representative sample of the Slovenian adult population via an online research panel. A total of 1189 participants (598 women), aged between 18 and 95 years (*M* = 46.7, *SD* = 16.2) completed the survey. Confirmatory and exploratory factor analyses, reliability analyses, hypothesis testing, and correlational analyses were conducted. The analyses rejected a hypothesised unidimensional model and demonstrated that a four-factor model with 27 items was the most theoretically and psychometrically adequate. The Slovenian version of Mental Health Literacy Scale (S-MHLS) consists of the following factors: (1) Attitudes Towards People With Mental Health Problems, (2) General Attitudes Towards Mental Health Problems and Help-Seeking, (3) Recognition of Mental Health Disorders and (4) Knowledge About Seeking Mental Health Information. The factors have adequate construct validity and internal consistency, which is also adequate for the entire S-MHLS. However, a decreased scope of the content might result in an inadequate representation of the construct of mental health literacy. In addition, the psychometric interpretation of the MHLS varies widely in validation studies across different linguistic contexts. Therefore, we propose a further improvement of the instrument with a psychometrically and theoretically sound multifactorial structure that demonstrates strong cross-cultural validity.

## Highlights

1. What do we already know about this topic? Mental health literacy has been recognized as a facilitator of help-seeking behaviour, thus, increasing mental health literacy is an important target of various interventions.
2. How does your research contribute to the field? This study represents a step forward in developing psychometrically sound mental health literacy measures, which currently remain scarce and underdeveloped.
3. What are your research’s implications towards theory, practice or policy? A psychometrically sound quantitative measure of mental health literacy is a useful and necessary tool for intervention evaluations and needs assessments in different populations.

## Introduction

Mental health literacy has been defined primarily as ‘knowledge and beliefs about mental disorders which aid their recognition, management or prevention’.^1^ Along with the original definition, Jorm and colleagues^1^ have proposed that mental health literacy consists of six distinct aspects, namely (1) recognition of mental health disorders, (2) knowledge about seeking mental health information, (3) knowledge about risk factors and causes, (4) knowledge about self-treatment, (5) knowledge about professional help available and (6) attitudes that promote recognition and appropriate help-seeking. This operational definition has been widely accepted. Later researchers proposed the inclusion of first aid mental health skills and underlined the role of knowledge and beliefs regarding prevention.^2^ In addition, they emphasised the role of positive mental health^3^ and help-seeking efficacy, referring to being ‘empowered to receive the best available help’.^4^

Regardless of the unreached consensus regarding the operational definition of mental health literacy, poor mental health literacy was identified as an important barrier to help-seeking.^5^ Improvement in this aspect was associated with reduced personal stigma^6^ and more positive beliefs and intentions about seeking professional help.^6–8^ In particular, positive attitudes regarding the treatment of mental health disorders are associated with more favourable attitudes towards help-seeking and a greater willingness to seek mental health services.^6,9^ Thus, improving mental health literacy is a goal of many interventions aimed at decreasing the treatment gap and better public mental health.^10^

The multitude of operational definitions of mental health literacy has been transferred onto a diverse spectrum of measurements. However, there remains a lack of comprehensive, robust, and psychometrically sound measurement tools.^3,11^ Many instruments focus exclusively on measuring literacy of specific mental health disorders, particularly depression, anxiety or schizophrenia.^1,12,13^ Some instruments exclusively measure specific domains of mental health literacy, including knowledge^14^ or positive mental health.^15^ The most commonly used measure of mental health literacy is the depression or schizophrenia vignette,^1^ which provides a comprehensive insight into the individual’s knowledge and attitudes about mental health and help-seeking. Assessment requires an individual approach and interpretation of qualitative responses.

To assess mental health literacy on a larger sample and in a more cost-effective manner, O’Connor and Casey^16^ developed the Mental Health Literacy Scale (MHLS). It is a robust and quantitative measure that covers the six aspects of mental health literacy identified by Jorm et al^1^ and relates to a variety of mental health disorders. The MHLS demonstrates adequate psychometric properties that suggest its use in the intervention/evaluation process.^16^

In recent years, additional validation studies have been published, suggesting the use of the MHLS in other linguistic and cultural contexts. Two studies examined the construct validity of the scale when translated into Farsi/Persian, resulting in modified versions of the scale.^17,18^ Kesgin et al^19^ explored the content and construct validity of the scale, as well as reliability, internal consistency and invariance of the scale in Turkish. The content validity of the scale was also examined in the South African and Zambian context.^20^

The present study aims to validate the Slovenian translation of the scale by thoroughly examining its psychometric properties on the general population of Slovenia. The study aims to assess construct validity through determining the factorial structure and assessing convergent and discriminant validity. It also aims to assess the scale’s internal consistency and cross-cultural validity.

## Material and Methods

The Commission for Ethics in Research of the Department of Psychology at the Faculty of Mathematics, Natural Sciences and Informational Technologies at the University of Primorska, Slovenia, evaluated that the study complied with the principles of ethics in research (application number 2020-04).

### Participants

A stratified sample of 1189 adult representatives of the general population was recruited. The sample consisted of 598 women (50.3%) and 591 men (49.7%), aged between 18 and 95 years (*M* = 46.7, *SD* = 16.2). The majority of the sample was employed (56.4%), having obtained a secondary education (50.9%). Participants were distributed representatively across all Slovenian regions and came from urban (58.2%) and rural (42.8%) environments.

### Procedures

This study is a web-based panel research that took place in February 2019. It was conducted by one of the largest private companies for online research in Slovenia, Valicon. Valicon holds a large database of panel members, who are divided into strata according to their gender, age and region. The number of selected participants in each stratum is proportionate to represent the general population. The participants in this study accepted the call to join after receiving an e-mailed invitation sent to all database individuals. Once each stratum was full, individuals were unable to respond to the invitation. No additional inclusion or exclusion criteria were specified.

Prior to starting the survey, participating members gave their informed consent. They then provided information about their socio-demographic profile and previous experience of mental health problems. They were also presented with a battery of questionnaires regarding mental health and mental health literacy. The battery of questionnaires was part of a larger project; only selected questionnaires were used in this study (see Measures). Participation in this research was rewarded with ‘participation points’ that recruited individuals could exchange for monetary awards once a year.

### Measures

Several questionnaires measuring mental health literacy and related constructs were adopted in the present study.

#### Mental Health Literacy Scale (MHLS).

The MHLS is a 35-item measure of mental health literacy.^16^ The content of the scale includes the six aspects of mental health literacy proposed by Jorm.^1^ The items that address the knowledge of mental health disorders were developed in accordance with the Diagnostic and Statistical Manual of Mental Disorders IV-TR^21^ and the Vignette Interview.^1^ The items employ various response formats, namely four-point Likert scales for likelihood (1 – *very unlikely*, 4 – *very likely*; 13 items) and helpfulness (1 – *very unhelpful*, 4 – *very helpful*; 2 items), and five-point Likert scales for agreement (1 – *strongly disagree*, 5 – *strongly agree*; 13 items) and willingness (1 – *definitely unwilling*, 5 – *definitely willing*; 7 items). The authors of the scale suggest a unidimensional structure, with scores ranging from 0 to 160. A high final score indicates a high level of mental health literacy. The scale exhibited adequate internal consistency (α = .873), good test-retest reliability and adequate content and structural validity.^16^ For the present study, the questionnaire underwent a blind back-translation process from English into Slovenian. First, a psychologist with excellent knowledge of English translated the instructions and all the items from English to Slovenian. Subsequently, an English expert translated the Slovenian version back to English. This version was additionally inspected by a second psychologist. The psychologists and the English expert then compared the two versions, discussed the discrepancies and jointly decided on a single most appropriate translation.

#### Attitudes Towards Depression (ATD).

Attitudes Towards Depression is a 30-item questionnaire, originally developed in the Slovenian language (orig. *Vprašalnik stališč do depresije*).^22^ The questionnaire measures depression-related literacy through assessing attitudes towards curing and healing of depression (α = .65), towards people suffering from depression (α = .64) and towards possible complications caused by depression (α = .63). Agreement with the items is assessed on a five-point Likert scale from 1 (*strongly agree*) to 5 (s*trongly disagree*). Final scores range from 30 to 150, with higher score indicating a more positive attitude towards depression and better depression-related literacy.

#### The Stigmatizing Attitudes-Believability (SAB).

A univariate scale with eight items was used to measure stigmatizing attitudes towards people with mental illness.^23^ The scale demonstrates adequate internal consistency (α = .78). A Likert scale is used, ranging from 1 (*not at all believable*) to 7 (*completely believable*). Final scores range from 8 to 56, with higher score indicating a more stigmatizing attitude. A process of blind back-translation (as described above) was applied to translate the questionnaire from English into Slovenian.

#### Knowledge of Available Sources of Professional Help.

Participants were asked to answer the following question: ‘Should you find yourself in a mental health crisis or distress, what professionals or services might you turn to or can you think of?’ Responses were rated in the following manner: (a) responses referring to formally recognised sources of help (e.g. doctors [GPs], psychiatrists, psychologists, psychotherapists, social workers, [psychiatric] clinics, social security centres, specific NGOs and telephone helplines) were rated with two points; (b) responses referring to sources of help that are not formally recognised but could potentially be helpful (e.g. priests, teachers, various alternative sources, friends, family and others) were rated with one point; (c) responses that did not specify any of the above-mentioned sources (e.g. ‘no one’, ‘I do not know’) were rated with zero points.

#### Demographic Variables.

Demographic information included gender, age, marital status, employment status, education, municipality, region, type of environment (urban vs. rural) and proximity to professional help. Finally, the participants reported their history of mental health problems and help-seeking, indicating a personal experience or an experience of someone in their close social circle (friends, relatives).

### Data Analysis

First, we determined the construct validity of the scale by testing the factorial structure of the MHLS.^24^ O’Connor and Casey^16^ suggested that the measure follows a unidimensional structure; therefore, we hypothesised that the translated instrument would retain its unidimensional form. We used confirmatory factor analysis (CFA) under the assumption that the covariance between all 35 indicators was explained by a single factor. A variance standardisation method was used to identify the model. To determine the fit of the model, the chi-square (*χ*^2^) statistics was applied, which assesses similarity between the estimated variance-covariance matrix and the sample variance-covariance matrix. A large sample size can potentially lead to spurious statistical significance; thus, we used alternative measures of fit with the corresponding cut-off values: the Comparative Fit Index (CFI) – .95, the Root Mean Square Error of Approximation (RMSEA) – .07, the 90% RMSEA confidence intervals (CI) – .08 and the Standardised Root Mean Square Residual (SRMR) – .08.^25^ Considering the heterogeneity of the scales, the SRMR may not be appropriate for our data.

Due to the insufficient psychometric properties of the unidimensional model, we furthermore conducted an exploratory factor analysis (EFA). Expecting correlations between factors, we chose direct oblimin rotation.^26^ To determine the number of factors, we used parallel analysis. We conducted a reliability analysis by calculating Cronbach’s α, McDonald’s ω and item-total correlations for each factor and for the entire Slovenian version of MHLS (S-MHLS).

We further determined construct validity through hypothesis testing.^24^ More specifically, we conducted a correlational analysis to examine the relationship of the S-MHLS factors with related constructs. We presented the correlations in the form of a matrix, which allowed us to determine the convergent and discriminant validity.

Statistical analyses were conducted using the R-based computer software Jamovi, version 1.2.2.

## Results

Descriptive statistics for the measures are presented in Table 1. For the MHLS and ATD, the participants did not reach the minimal and maximal scale scores. For the SAB, these scores were reached. There were instances where participants did not report any relevant sources of professional help. The Cronbach’s α values were satisfactory for all measures, except for the Knowledge of Help Sources, which is a single -item measure.^26^

**Table 1.** Descriptive statistics for the measures used.

	Min	Max	M	SD	*SE*	Cronbach’s α
MHLS (35 items)	77	154	114.09	11.65	.34	.84
SAB	8	56	24.42	7.74	.22	.80
ATD	73	146	107.69	11.80	.34	.77
Knowledge of Help Sources	0	17	3.85	2.42	.07	N/A

*Note:* MHLS = Mental Health Literacy Scale; SAB = Stigmatizing Attitudes-Believability; ATD = Attitudes Towards Depression.

### Exploring Factorial Structure and Internal Consistency

We applied a CFA to test the hypothesised unidimensional structure. In the one-factor model, the standardised loading estimates of most items were statistically significant, except for two items (no. 9 and no. 15). However, the statistically significant loading estimates were low, ranging from .09 to .77 (*M* = .35, *SD* = .20), with only seven of them (nos. 24, 29–33, 35) exceeding the suggested threshold of .5.^26^ This suggests that it is unlikely that all items in this scale measure the same latent construct. To examine how the hypothesised model fits the data, we used several measures of fit. A chi-square test indicated a discrepancy between the estimated variance-covariance matrix and the sample variance-covariance matrix, χ^2^ (5894, *N* = 1189), P < .001. This indicates that the model is not a good fit. A similar conclusion can be drawn from a low CFI value of .49 and a relatively high RMSEA value of .09 (90% CI ≈ .09). Finally, the poor fit of the model is attributed to the high SRMR value of .09. Considering the low loading estimates and insufficient values of the fit indices, we concluded that the Slovenian version of the MHLS does not follow a unidimensional factorial structure.

Therefore, we conducted an EFA with principal axis extraction and direct oblimin rotation. We included all 35 items of the MHLS questionnaire. The calculated Kaiser–Meyer–Olkin measure was .88, which confirmed sample adequacy for the analysis. Bartlett’s test of sphericity (*χ*^2^ (595) = 11.00, P < .001) indicated that the correlations between items are large enough for the EFA. The factor loadings of the relevant items are presented in Table 2.

**Table 2.** The results of exploratory factor analysis.

Item	MHLS factors			
	1 Attitudes Towards People	2 General Attitudes	3 Recognition	4 Information Seeking
1 Recognition of social phobia			**.53**	
2 Recognition of generalized anxiety disorder			**.58**	
3 Recognition of major depressive disorder			**.52**	
4 Recognition of personality disorders			**.47**	
5 Recognition of dysthymia			**.50**	
6 Recognition of agoraphobia			**.49**	
7 Recognition of bipolar disorder			**.47**	
8 Recognition of drug dependence			**.45**	
16 Information seeking – where to seek info				**.53**
17 Information seeking – using a computer or a telephone				**.38**
18 Information seeking – face to face				**.38**
19 Information seeking – access to resources				**.73**
21 Mental illness is a sign of weakness		**.53**		
22 Mental illness is not a real medical illness		**.52**		
23 People with a mental illness are dangerous		**.49**		
24 It is best to avoid people with a mental illness		**.53**		
25 If I had a mental illness I would not tell anyone		**.55**		
26 Seeing a professional means you are not strong enough		**.62**		
27 If I had a mental illness, I would not seek help		**.63**		
28 I believe treatment for a mental illness would not be effective		**.71**		
29 Willingness to move next door to sb. with mental illness	**.69**			
30 Willingness to spend an evening with sb. with mental illness	**.85**			
31 Willingness to make friends with sb. with mental illness	**.84**			
32 Willingness to work closely with sb. with mental illness	**.71**			
33 Willingness to have sb. with m. illness marrying into family	**.52**			
34 Willingness to vote for politician if you knew they had m. illness	**.29**			
35 Willingness to employ sb. if you know they had a mental illness	**.46**			
Eigenvalues	5.48	2.14	1.94	.83
% of variance	9.75	8.85	7.22	4.18
Cronbach’s α	.87	.83	.75	.59
McDonald’s ω	.87	.83	.75	.63
*M (SD)*	23.54 (4.81)	32.78 (5.59)	23.69 (3.33)	15.11 (2.34)
*Min–max*	7–35	10–45	8–32	6–20
Skewness *(SE)*	−.12 (.07)	−.34 (.07)	−.61 (.07)	−.15 (.07)
Kurtosis *(SE)*	.41 (.14)	.20 (.14)	1.40 (.14)	.21 (.14)
K–S test	.05^***^	.10^***^	.06^***^	.11^***^

*Note*. Attitudes towards people = Attitudes Towards People With Mental Health Problems; General attitudes = General Attitudes Towards Mental Health Problems and Help-Seeking; Recognition = Recognition of Mental Health Disorders; Information Seeking = Knowledge About Seeking Mental Health Information; K-S test = Kolmogorov–Smirnov test of normality of distribution; sb. = somebody/someone; m. = mental.

*P < .05, ** P < .01, *** P < .001.

**Figure A1.**
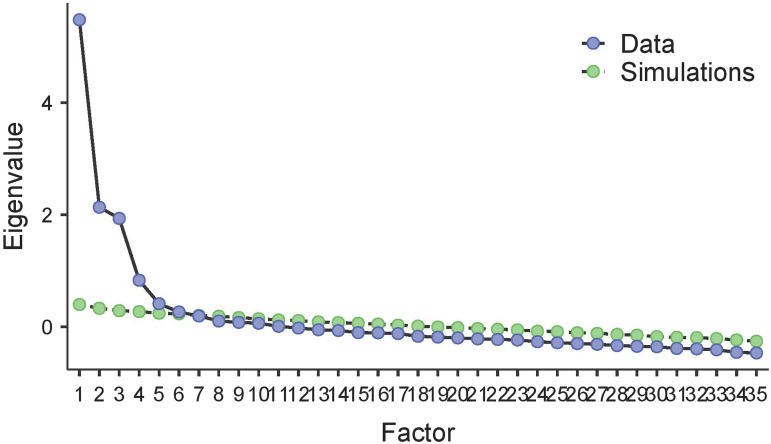
Scree plot for exploratory factor analysis.

The EFA using the parallel analysis criterion indicated an initial structure of six factors. Upon further inspection, the last two factors were excluded. The factor loadings of the items composing the two factors (*M*_5_ = .25, *M*_6_ = .22), the share of the common variance explained by these factors (2.96% and 2.39%, respectively), and their eigenvalues (.41 and .26, respectively) were low. Additionally, we inspected a scree plot (see Figure A1, Appendix), which confirmed that four factors are sufficient.^26^Moreover, the content of the items loading these two factors (nos. 5–6) does not reflect a theoretically grounded latent construct. This is in contrast to the first four factors (see Table 2), which form theoretically meaningful constructs, namely (1) Attitudes Towards People With Mental Health Problems, (2) General Attitudes Towards Mental Health Problems and Help-Seeking, (3) Recognition of Mental Health Disorders and (4) Knowledge About Seeking Mental Health Information. In addition, the factor loadings of the items that load predominantly on these factors are adequate (see Table 2) and meet the minimum criterion for interpretation (±.30).^26^ The exceptions that do not meet this criterion are discussed below. The first three factors meet the eigenvalue criterion of 1,^26^ which is not met by the fourth factor. However, removing this factor would result in a theoretically restricted questionnaire with only two aspects of mental health literacy included. Furthermore, excluding the fourth factor would reduce the already low cumulative variance of 29.99%.

To define the factors, we further examined the EFA results and analysed the scales’ coefficients of internal consistency by calculating Cronbach’s α and McDonald’s ω (scale and scale if item deleted), as well as item-rest correlations. The content of all the items composing factor no. 1 reflects the same latent construct, namely Attitudes Towards People With Mental Health Problems. Deleting any of the items in factor no. 1 would not result in higher α or ω. The item-rest correlations range from .51 (item no. 33) to .72 (item no. 31). Item no. 33, namely ‘Willingness to vote for a politician if you knew they had suffered a mental illness’, has the lowest item-rest correlation and factor loading; however, its deletion does not change the reliability values (Cronbach’s α or McDonald’s ω). Moreover, item no. 33 does not appear to theoretically diverge from other items included in this factor, measuring attitudes towards people with mental health problems. Therefore, we propose to retain this item. The content of the items included in factor no. 2 reflects a latent construct General Attitudes Towards Mental Problems and Help-Seeking. For this factor, item-rest correlations range from .44 (item no. 25) to .61 (item no. 21), with the exception of item no. 20, namely ‘People with mental illness could snap out of it if they wanted’ (rest-item correlation of .25). Deleting this item would result in higher α and ω of .83 (for both measures). Although the item is theoretically more associated with factor no. 2, it also loads factor no. 4 (−.31). This item could be problematic partially due to translation issues. Upon closer inspection, we noticed that the Slovenian translation of the item lacks the negative connotation that is present in the original; it could have a double meaning and thus be understood differently by participants than in the original. Therefore, we propose to exclude this item when applying the instrument. The content of all the items included in factor no. 3 reflects the same latent construct, namely Recognition of Mental Health Disorders. For factor no. 3, deleting any of the items would not result in a higher α or ω. The corrected item-total correlations range from .39 (item no. 5) to .49 (item no. 4). The content of the items included in factor no. 4 refers to the factor named Knowledge About Seeking Mental Health Information. The item-rest correlations for this factor range from .25 (item no. 17) to .54 (item no. 19). Item no. 17, namely ‘Information seeking – using a computer or a telephone’, has the lowest item-rest correlation. Deleting this item would result in higher α of .63 and ω of .64. The item could be problematic because it refers to the usage of phones and computers when seeking mental health information, which may not be equally available to people from certain socio-demographic backgrounds. However, the factor loading value for this item is acceptable^20^ and the content of this item reflects the same latent construct as the remaining items in factor no. 4. In addition, factor no. 4 consists of only four items, thus deleting one item would result in a lack of items in the factor, jeopardising the adequate representation of the construct. Therefore, we suggest retaining item no. 17.

The original MHLS version contains additional seven items that were excluded due to poor psychometric properties. The content of these items relates to knowledge of mental health problems’ risk factors and causes, knowledge of self-treatment and knowledge of available treatments. Some of these items predominantly load factor no. 3; however, their factor loadings are lower than the factor loadings of the items referring to recognition of mental health disorders. In addition, some of these items also load factor nos. 5–6. Their factor loadings were low (.21–.32) and dispersed, and did not form any theoretically grounded construct.

The distributions of scores are negatively skewed for all four factors, meaning that the frequent scores are clustered at the high end of the distribution. The magnitude of skewness varies and is greatest for factor no. 3. All four factors have positive kurtosis, meaning that they have many scores at the tails and contain outliers. The value of kurtosis varies and is largest for factor no. 3. Kolmogorov-Smirnov test identified statistically significant deviations from normal distribution for all four factors.

Considering the results, the four-factor model with 27 items proves to be the most viable. All four factors demonstrate sufficient internal consistency as measured by Cronbach’s α and McDonald’s ω (see Table 2). These values are also satisfactory for the entire S-MHLS (Cronbach’s α = .85, McDonald’s ω = .85) with 27 items. The Cronbach’s α value of the S-MHLS exceeds the Cronbach’s α value of the original MHLS (see Table 1), indicating that the new multifactorial structure demonstrates increased reliability.

### Assessing Construct Validity Through Correlational Analysis

To assess the construct validity of the factors, we analysed correlations between the S-MHLS factors and other constructs from the mental health literacy domain. The correlations are presented in Table 3.

**Table 3.** Correlation matrix for assessing convergent and discriminant validity.

	MHLS factors							
	Attitudes Towards People	General Attitudes	Recognition	Information Seeking	S-MHLS	ATD	SAB	Knowledge of Help Sources
Attitudes Towards People	-							
General Attitudes	.34^***^	-						
Recognition	.19^***^	.19^***^	-					
Information Seeking	.25^***^	.26^***^	.25^***^	-				
MHLS	.73^***^	.78^***^	.54^***^	.53^***^	-			
ATD	.38^***^	.70^***^	.21^***^	.33^***^	.68^***^	-		
SAB	−.34^***^	−.43^***^	−.03^***^	−.13^***^	−.40^***^	−.44^***^	-	
Knowledge of Help Sources	.21^***^	.27^***^	.17^***^	.21^***^	.32^***^	.27^***^	−.14^***^	-

*Note.* Reported correlation coefficients: Spearman for ‘Knowledge of Help Sources’, Pearson for other variables; ATD = Attitudes Towards Depression; SAB = The Stigmatizing Attitudes-Believability; S-MHLS = Slovenian version of the Mental Health Literacy Scale (27 items).

*P < .05, ** P < .01, *** P < .001.

We assessed the discriminant validity of the factors based on their inter-correlations. Low correlations, as presented in Table 3, indicate adequate discriminant validity and confirm the uniqueness of each factor. The statistical significance of these correlations can be attributed to the large sample size.

To assess the convergent validity of the factors, we examined correlations of the S-MHLS factors with other measures of related constructs. The first evidence of convergent validity is the positive correlation between the ATD (measuring depression-related attitudes and literacy) and all S-MHLS factors. Given the ATD’s focus on the depression-related attitudes, the particularly strong correlation between the ATD and the two factors refering to mental health related attiudes provides additional evidence of the convergent validity of these two factors. The convergent validity of the two factors is further supported by the relatively strong negative correlation with the SAB (measuring stigmatizing attitudes). The less pronounced correlations between the SAB and the other two factors (Recognition and Information Seeking) suggest a stronger discriminant validity of these factors. Finally, knowledge of sources of professional help is another indicator of mental health literacy. This measure was included to test the hypothesis that individuals who listed more sources of help have higher mental health literacy. The identified correlations with all S-MHLS factors provide evidence of construct validity for all factors.

## Discussion

The Slovenian version of the MHLS (S-MHLS) contains 27 items forming four theoretically and psychometrically sound factors, corresponding to three aspects of mental health literacy.^1^ Two factors, Attitudes Towards People With Mental Health Problems and General Attitudes Towards Mental Health Problems and Help-Seeking, refer to attitudes that promote acceptance of mental health problems and help-seeking. This definition is particularly applicable to the latter factor, whereas the former concerns one’s willingness to interact with people with mental illness. Both types of attitudes are related to stigmatizing beliefs about mental illnesses. The third factor, Recognition of Mental Health Disorders corresponds to the aspect of knowledge about symptoms of common mental health disorders. The forth factor, Knowledge About Seeking Mental Health Information, refers to knowledge of where and how to seek for mental health information. The identified four factors show adequate construct validity and reliability. The latter is also adequate for the entire S-MHLS questionnaire.

The four-factor model results in a reduced scope of the original MHLS content. The three aspects of mental health literacy are not included; risk factors and causes, knowledge of self-treatment and knowledge of available professional help. The items representing these aspects are scarce and show an unclear factorial structure. To ensure that the measure covers all the aspects of the construct, we suggest including more theoretically and psychometrically supported items. Further development of the instrument may lead to a factorial structure that reflects all theoretically proposed aspects of mental health literacy. Mapping the aspects into the factorial structure provides conformation that these aspects are adequately captured in the measurement. Considering the practical application of the instrument, the ability to measure the specific aspect of mental health literacy proves necessary. When using the measurement for intervention evaluation, identifying the aspects that show the greatest change is valuable feedback that helps to identify the specific strengths and weaknesses of the intervention.

All four factors show a skewed distribution. On the one hand, this suggests that the level of mental health literacy in the population is relatively high. On the other hand, it may indicate that the instrument does not sufficiently differentiate between people with better mental health literacy. The most prominent deviation from the normal distribution, as present in factor no. 3, may be attributed to the potentially suggestive wording of the questions in this factor.

Psychometric interpretations of the MHLS vary widely in validation studies across different linguistic contexts, suggesting either a unidimensional instrument^18^ or an instrument with a multifactorial structure.^17^ Heizomi et al^17^ proposed a 30-item five-factor model, slightly reducing the scope of the original scale. In contrast, Nejatan et al^18^ proposed a unidimensional but modified version of the MHLS with 29 items. Interestingly, both interpretations were conducted in the Farsi/Persian language. A unidimensional structure, resembling the original scale, has been proposed in Turkish,^19^ South African and Zambian context.^20^ Thus, the psychometric interpretation of this scale appears to be dependent on the validation procedure and the cultural/linguistic content, which suggests a rather questionable cross-cultural validity. This confirms the above-mentioned argument regarding the need to develop a more psychometrically sound measure of mental health literacy with a factorial structure that is consistent across different linguistic contexts.

Given the complexity of the construct of mental health literacy and the overlap of this construct with other well-researched constructs (e.g. stigmatising attitudes), Spiker and Hammer^27^ suggested that mental health literacy is not only a construct, but a theory. Considering mental health literacy as a theory would allow the researchers to explore the relationship between these constructs and identify the role that particular constructs play in help-seeking behaviour. To identify these relations, a multifactorial structure of the measure is required.

The present study has two major limitations. Notwithstanding the large representative sample, only panel members are included in the recruitment of participants, making random selection from the population impossible. This potential sampling bias may lead to reduced external validity and limit the generalisation of the results. We recommend testing the factorial structure of the measure on other samples. The lack of a ‘gold standard’, psychometrically sound instruments, and problems with operationalising mental health literacy limit the validity of the analysis and hinder a critical review of the results.

## Conclusions

This study aimed to validate a Slovenian translation of the MHLS through a comprehensive examination of its psychometric properties. In contrast to the original unidimensional scale with 35 items, the results of the present study revealed a 27-item scale (S-MHLS) with four factors that demonstrate a reliable internal consistency and adequate convergent and discriminant validity. However, due to excluding certain items, the proposed four-factor model leads to a weak representation of certain aspects of mental health literacy. We propose to improve the scale’s content, following the theoretical predispositions of mental health literacy and to achieve the desired psychometric properties that are consistent across different linguistic and cultural contexts. Considering a branched operational definition of mental health literacy, we argue that a multifactorial structure of the measure is necessary to operationalize its’ aspects and provide a useful diagnostic tool for intervention planning or evaluations. Therefore, this study is a step forward in the development of theoretically and psychometrically sound measure of mental health literacy.
